# In Silico Evaluation of Coding and Non-Coding nsSNPs in the Thrombopoietin Receptor (*MPL*) Proto-Oncogene: Assessing Their Influence on Protein Stability, Structure, and Function

**DOI:** 10.3390/cimb45120589

**Published:** 2023-11-23

**Authors:** Hakeemah H. Al-nakhle, Hind S. Yagoub, Sadin H. Anbarkhan, Ghadah A. Alamri, Norah M. Alsubaie

**Affiliations:** 1 Department of Clinical Laboratory Sciences, College of Applied Medical Sciences, Taibah University, Al-Madinah Al-Monawarah 42353, Saudi Arabia; hundsay@gmail.com (H.S.Y.); sadin.ak12@gmail.com (S.H.A.); no8ra18.asub@gmail.com (N.M.A.); 2 Faculty of Medical Laboratory Sciences, Omdurman Islamic University, Omdurman 14415, Sudan

**Keywords:** nsSNPs, *MPL*, hematopoiesis, hematological disorders, in silico analysis, protein structure and function, coding, non-coding

## Abstract

The thrombopoietin receptor (*MPL*) gene is a critical regulator of hematopoiesis, and any alterations in its structure or function can result in a range of hematological disorders. Non-synonymous single nucleotide polymorphisms (nsSNPs) in *MPL* have the potential to disrupt normal protein function, prompting our investigation into the most deleterious *MPL* SNPs and the associated structural changes affecting protein–protein interactions. We employed a comprehensive suite of bioinformatics tools, including PredictSNP, InterPro, ConSurf, I-Mutant2.0, MUpro, Musitedeep, Project HOPE, STRING, RegulomeDB, Mutpred2, CScape, and CScape Somatic, to analyze 635 nsSNPs within the *MPL* gene. Among the analyzed nsSNPs, PredictSNP identified 28 as significantly pathogenic, revealing three critical functional domains within *MPL*. Ten of these nsSNPs exhibited high conservation scores, indicating potential effects on protein structure and function, while 14 were found to compromise *MPL* protein stability. Although the most harmful nsSNPs did not directly impact post-translational modification sites, 13 had the capacity to substantially alter the protein’s physicochemical properties. Some mutations posed a risk to vital protein–protein interactions crucial for hematological functions, and three non-coding region nsSNPs displayed significant regulatory potential with potential implications for hematopoiesis. Furthermore, 13 out of 21 nsSNPs evaluated were classified as high-risk pathogenic variants by Mutpred2. Notably, amino acid alterations such as C291S, T293N, D295G, and W435C, while impactful on protein stability and function, were deemed non-oncogenic “passenger” mutations. Our study underscores the substantial impact of missense nsSNPs on *MPL* protein structure and function. Given *MPL*’s central role in hematopoiesis, these mutations can significantly disrupt hematological processes, potentially leading to a variety of disorders. The identified high-risk pathogenic nsSNPs may hold promise as potential biomarkers or therapeutic targets for hematological diseases. This research lays the foundation for future investigations into the *MPL* gene’s role in the realm of hematological health and diseases.

## 1. Introduction

The *MPL* gene, located on chromosome 1p34, is responsible for encoding the receptor for thrombopoietin, a pivotal hematopoietic growth factor. Structurally, the *MPL* gene consists of 12 exons. Importantly, its two ‘cytokine receptor domains’ are each represented by four exons. One exon designates the transmembrane region, while two others define the cytoplasmic domain. The protein product of the *c-mpl* gene, CD110, is made up of 635 amino acids. This protein features a transmembrane domain, two outer cytokine receptor domains, and two inner cytokine receptor box motifs [1].

The *MPL* receptor is crucial for the proliferation of megakaryocytes, the cells that generate platelets essential for blood coagulation [2]. There is evidence to suggest that the thrombopoietin receptor also has a role in preserving hematopoietic stem cells [3]. These stem cells, found in bone marrow, can evolve into red and white blood cells, as well as platelets. The thrombopoietin receptor becomes active upon binding with the thrombopoietin protein. Once activated, it triggers the JAK/STAT signaling pathway, a critical process for regulating blood cell production, by relaying chemical messages from the cell’s exterior to its nucleus [4].

While TPO’s primary role is in hematopoiesis, there is evidence to suggest that TPO-MPL signaling can indirectly promote angiogenesis, primarily through its influence on hematopoietic stem cells and the bone marrow microenvironment [5]. TPO-MPL signaling can lead to the mobilization of hematopoietic stem cell (HSCs) from the bone marrow into the peripheral blood [5]. This process is important for replenishing blood cell populations, including platelets, in response to increased demand.

HSCs have the potential to contribute to angiogenesis through several mechanisms. They can differentiate into endothelial progenitor cells (EPCs), which are precursors of endothelial cells—the primary cells that makeup blood vessels [6]. These EPCs can be incorporated into newly forming blood vessels and promote angiogenesis [7]. TPO-MPL signaling may stimulate the production of vascular endothelial growth factor (VEGF), a potent angiogenic factor [8]. VEGF is crucial for the formation and maintenance of blood vessels. Increased VEGF levels can lead to angiogenesis by promoting endothelial cell proliferation, migration, and vessel permeability.

TPO-MPL signaling leads to the production and release of platelets from megakaryocytes. Platelets play a multifaceted role in angiogenesis. They can store and release pro-angiogenic factors like platelet-derived growth factor (PDGF), fibroblast growth factor (FGF), and VEGF, which can stimulate endothelial cell activation and angiogenic processes [9]. Moreover, TPO-MPL signaling also influences erythropoiesis (red blood cell production). Increased red blood cell production can improve oxygen delivery to tissues [10], including those undergoing angiogenesis, thereby facilitating the formation of new blood vessels.

*MPL* mutations, primarily in exon 10, are linked to myeloproliferative neoplasms (MPNs) like essential thrombocythemia (3%) and primary myelofibrosis (5%), as well as hereditary thrombocytosis. These mutations, especially *MPL* W515 variants, show a gain-of-function (GOF) activity, often leading to the receptor’s constitutive activation. The W515 position in *MPL*, crucial for preventing receptor dimerization, undergoes substitutions causing significant thrombocytosis in models. Other mutations outside exon 10, with lower GOF than W515, also exist and may combine with events like *JAK2* mutations to produce disease phenotypes. The *MPL* S505N mutation, found in familial and sporadic cases, causes continuous *MPL* activation [11].

Another *MPL* mutation, specifically *MPL* P106L and *MPL* K39N, discovered in hereditary thrombocytosis, is associated with high thrombopoietin (TPO) levels, setting them apart from other *MPL* mutations. *MPL* K39N, prevalent in 7% of African Americans, leads to mild thrombocytosis in heterozygous individuals and more pronounced effects in homozygous cases, likely due to its low cellular expression suggesting a loss-of-function (LOF) mutation. *MPL* P106L, first discovered in an Arab family, is associated with strong thrombocytosis, especially in homozygous individuals. While initially believed to be faulty in cell surface translocation yet able to bind TPO, further research indicated its LOF nature, with a limited presence on the cell surface in megakaryocyte (MK) progenitors and a role in both MK proliferation and disrupted TPO clearance. The mouse model confirmed its association with thrombocytosis and elevated TPO levels [11].

Single nucleotide polymorphisms (SNPs) represent the most prevalent genetic variations in humans, with an estimated 3–5 million instances [12]. They refer to variations where just one nucleotide differs between individuals. When SNPs occur in coding areas, they can modify amino acid sequences, leading to changes in protein functions; these are called non-synonymous SNPs (nsSNPs). Research confirms that these alterations can manifest as observable traits [12,13,14]. Half of all SNPs fall under the category of nsSNPs, which can influence a protein’s structure and functionality. Furthermore, mutations in the intricately organized non-coding regions of a gene can significantly affect gene expression. Changes in the 5′ and 3′ untranslated areas can shift the protein’s secondary structure, subsequently influencing the attachment of proteins and ligands to these regions [15].

The human genome comprises a vast array of genetic polymorphisms, necessitating extensive research to understand each variant’s relevance, especially concerning disease predisposition and tailored drug design. To streamline this immense task, many computational methods have been introduced to pinpoint potentially significant variants for further in vitro or in vivo testing. Using specific algorithms, in silico techniques offer an efficient way to differentiate harmful SNPs from benign ones. Various databases also enable the examination of the holistic impact of polymorphisms, including their functional and structural implications.

In this study, we leverage publicly available datasets and open-access bioinformatics tools to pinpoint the most detrimental nsSNPs in the *MPL* gene and examine their effect on coding and non-coding regions. While previous in silico analyses have examined nsSNPs in the *MPL* gene linked to hematological disorders [16], comprehensive evaluations of their influence on protein structure, function, and post-translational changes are lacking. Our study not only seeks to define the structural outcomes but also to establish the correlation between specific nsSNPs and their oncogenic potential.

## 2. Materials and Methods

### 2.1. Retrieval of nsSNPs from the Database

**Figure 1:**
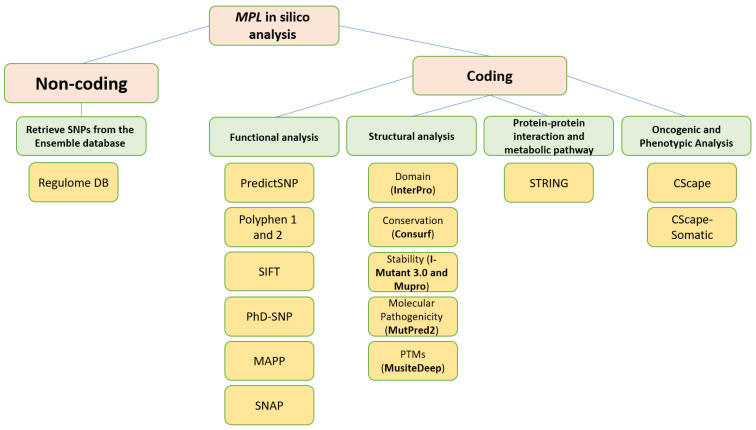
Illustration of the entire methodological approach.

The nsSNPs dataset for the *MPL* gene was obtained from the ENSEMBL database [17], and its accession number is ENSG00000117400. To identify a missense nsSNP associated with this gene, a missense filter was applied. The protein sequence corresponding to the *MPL* gene, in FASTA format, was retrieved from the NCBI database using the ID NP_005364.1. A visual representation of the entire methodological approach is provided in Figure 1.

### 2.2. Determining the Most Deleterious SNPs

To determine whether a given SNP is likely deleterious or benign, we utilized PredictSNP [18]. This tool offers predictions on the functional implications of the nsSNPs sourced from the ENSEMBL database (https://loschmidt.chemi.muni.cz/predictsnp, accessed on 21 December 2022). Essentially, PredictSNP harnesses the power of multiple prediction tools, amalgamating their results to deliver a comprehensive and integrated perspective on the potential effects of an SNP. Specifically, PredictSNP integrates outputs from tools including SIFT, PolyPhen-1 and PolyPhen-2, MAPP, PhD-SNP, and SNAP. This collective approach ensures PredictSNP offers a broader insight and achieves greater accuracy than standalone predictors.

### 2.3. Identification of nsSNPs within the Domains of the MPL Gene

To identify the locations of nsSNPs within the conserved domains of *MPL*, we utilized the InterPro tool (https://www.ebi.ac.uk/interpro/; accessed on 21 December 2022) [19]. InterPro amalgamates various databases, including Pfam, PROSITE, PRINTS, ProDom, SMART, TIGRFAMs, PIRSF, SUPERFAMILY, Gene3D, and PANTHER. This integration allows it to recognize protein motifs and domains, facilitating the functional characterization of a protein [20]. For this analysis, the protein sequence should be provided as input in FASTA format.

### 2.4. Assessment of the Conservation Profile Using ConSurf

To pinpoint potential functional and structural amino acids and evaluate their evolutionary conservation trajectory, we employed Consurf (https://consurf.tau.ac.il/, accessed on 21 December 2022) [21,22], an online tool that leverages the Bayesian method to study the evolutionary tendencies of amino acids. The conservation ratings were projected onto the target structure and denoted using Consurf’s color scale, where shades from cyan to purple represent grades from 1 (most variable) to 9 (highly conserved evolutionarily).

### 2.5. Prediction Alterations in Protein Stability

To examine the impact of amino acid changes on protein stability, we utilized two online tools: I-Mutant 3.0 and MUpro. I-Mutant 3.0 can be accessed at http://gpcr2.biocomp.unibo.it/cgi/predictors/I-Mutant3.0/I-Mutant3.0.cgi (accessed on 21 December 2022) and is an Support Vector Machine (SVM) -based tool designed to predict if a given amino acid alteration will lead to the stabilization or destabilization of a protein. Missense nsSNPs were assessed at 25 °C with a pH of 7.0. Subsequent analysis centered on the ΔΔG value and binary classification. Additionally, each result was supplemented with a reliability index (RI) value [23].

MUPro (http://mupro.proteomics.ics.uci.edu/, accessed on 21 December 2022) [24] was also employed to evaluate protein stability. This tool uses two machine-learning techniques, SVM and neural networks, both trained on an extensive mutation dataset. Notably, MUPro can predict changes in stability without the need for a protein’s tertiary structure. The tool’s efficacy was verified with a dataset comprising 1615 mutations, undergoing 20-fold cross-validation, achieving an accuracy of 84.2% when only sequence data was inputted. The outcome displays the energy shift arising from an amino acid substitution, denoted as a ΔΔG value, and a confidence score ranging from −1 to 1. Predictions were based on default settings, with interpretation mirroring the previously outlined method. A confidence score below 0 suggests the mutation diminishes protein stability, while a score above 0 implies enhanced stability due to the mutation.

### 2.6. Identification of MPL’s Post-Translational Modification Sites Using MusiteDeep

MusiteDeep is a tool designed to visualize post-translational modifications (PTMs) sites within protein sequences. It analyzes post-translational modifications, including phosphorylation, glycosylation, ubiquitination, sumoylation, acetyl-lysine, methylation, pyrrolidone carboxylic acid, palmitoylation, and hydroxylation. For this study, the thrombopoietin protein sequence, provided in FASTA format, was utilized as the input for MusiteDeep [25].

### 2.7. Predicting Protein–Protein Interactions Using the Search Tool for the Retrieval of Interacting Genes/Proteins (STRING)

Protein–protein interactions play a crucial role in ensuring the correct regulation and functionality of a protein’s biological activity. The STRING database, accessible at http://string-db.org (accessed on 21 December 2022), aids in uncovering intricate relationships between proteins. As of now, the STRING library encompasses 24,584,628 proteins spanning 5090 organisms. For our analysis, we provided the FASTA sequence of the *MPL* protein as input. The resulting data showcases all anticipated interactions, each coupled with a confidence score.

### 2.8. Analysis of the Functional Relevance of Non-Coding SNPs (ncSNPs) in the MPL Gene

We scrutinized the non-coding regions of the *MPL* gene using the ENSEMBL database. SNPs from the 5′ and 3′ regions were filtered out, and a selection criterion of a minor allelic frequency (MAF) below 0.001 was set. Using Regulome DB [26], these SNPs were linked to regulatory elements in the human genome. Input specifics: SNP IDs with MAF <0.001 (from 5′ or 3′ UTRs) obtained from the Ensemble database. The output details included chromosome location, dbSNP IDs, rank, and score. It is pivotal to identify functional variants and comprehend their implications. Notably, variants outside the protein-coding regions can introduce potential regulatory modifications. RegulomeDB facilitates the identification and assessment of SNPs predicted to operate as regulatory elements. The platform ranks SNPs based on the RegulomeDB score, which amalgamates data from several comprehensive databases, including ENCODE ChIP-seq, FAIRE, DNase I hypersensitive sites, eQTLs, and dsQTLs.

Guided by this scoring schema, SNPs were categorized into six distinct ranks based on their anticipated influence on transcription factor binding or gene expression regulation:

- Rank 1: strong evidence suggesting potential to affect binding and is associated with the expression of a gene target;
- Rank 2: likely to influence binding;
- Rank 3: lesser likelihood of affecting binding;
- Ranks 4, 5, 6: limited evidence of binding and are typically void of known regulatory functions.

### 2.9. Assessment of the Structural Impact of nsSNPs on the Human MPL Protein

To ascertain the potential structural consequences of nsSNPs on the human *MPL* protein, we employed Project HOPE (Project Have Your Protein Explained; available at https://www3.cmbi.umcn.nl/hope/, accessed on 21 December 2022) [27]. This server requires protein sequences in FASTA format and detailed SNP data as its input.

Project HOPE operates by collating and analyzing data from various databases and web servers, including the likes of WHAT IF, UniProt, and Distributed Annotation System (DAS). Consequently, it offers a homology model that aids in better comprehending the effects of mutations. The comprehensive report generated by Project HOPE elucidates the potential implications of SNPs on both the structure and function of the protein, contrasting wild-type and mutant versions. This report is complemented with pertinent annotations, visualizations, and animated representations.

### 2.10. Evaluation of Molecular Pathogenicity of nsSNPs

To assess the potential molecular repercussions of amino acid substitutions in the *MPL* protein, we utilized MutPred2 (available at http://mutpred.mutdb.org/index.html, accessed on 21 December 2022). This tool gauges the likelihood of amino acid replacements leading to either loss or gain of specific phenotypic effects on proteins, operating at a *p*-value threshold of 0.05 [28]. MutPred2 incorporates various structural and functional attributes in its analyses, encompassing aspects like secondary structure, transmembrane topology, signal peptide, catalytic functionality, binding tendencies (both macromolecular and metallic), PTMs, and allosteric effects.

Amino acid modifications can instigate an array of functional shifts in proteins. These alterations can span disruptions in protein stability and architecture, impediments in macromolecular partnerships, removal of PTM sites, and more. These molecular adjustments can culminate in phenotypic variations in protein behavior. With the assistance of the MutPred2 algorithm, pathogenicity levels associated with nsSNPs are dissected. This analysis offers a comprehensive picture of the possible structural and functional changes due to amino acid alterations. For each such alteration, both posterior probabilities and empirical *p*-values are derived.

For our assessment, the MutPred2 platform was furnished with the FASTA sequence of the human *MPL* protein, alongside detailed data on single amino acid changes. MutPred2’s output encapsulates an overarching probability indicating the potential deleterious or disease-associated nature of the amino acid shift. This output also includes a ranked compilation of specific molecular deviations that might influence the phenotype, each partnered with its respective *p*-value, adhering to the <0.05 threshold.

### 2.11. Oncogenic and Phenotypic Analysis

The oncogenic potential of the pinpointed nsSNPs was scrutinized using the CScape and CScape Somatic tools. CScape assigns each nsSNP a quantified score, subsequently categorizing them as either oncogenic with high confidence, malignant, or benign. In coding regions, these high-confidence predictions demonstrate an accuracy rate of approximately 92%, while in non-coding regions, the accuracy rate is about 76%. Conceptually, a greater score is indicative of a more pronounced oncogenic trait [29]. Moreover, the CScape Somatic tool is specifically designed to anticipate the oncogenic nature (either disease-driving or neutral) of somatic point mutations present in both coding and non-coding sections of the cancerous genome [30].

## 3. Results

### 3.1. Identification of Deleterious nsSNPs

To pinpoint the nsSNPs with the potential to profoundly impact the structure or functionality of the *MPL* protein, we deployed a suite of six distinct in silico nsSNP prediction tools, including PredictSNP, MAPP, PhD-SNP, PolyPhen1, PolyPhen2, SIFT, and SNAP. Out of the 635 evaluated nsSNPs, a consensus among all the tools identified 28 nsSNPs as deleterious (Table 1).

### 3.2. Identification of MPL Gene Domains Using InterPro

**Figure 2:**
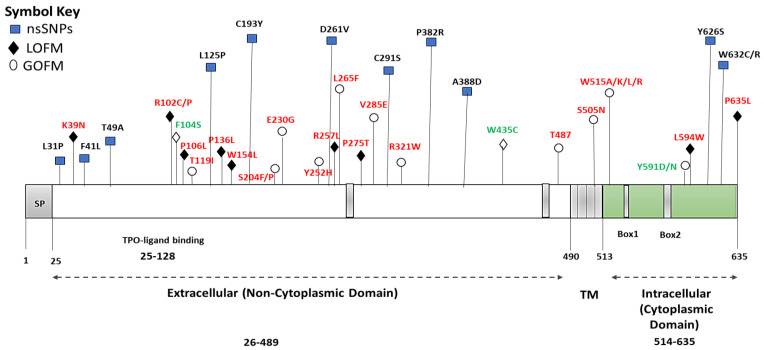
Domain mapping of the *MPL* protein via InterPro Server. This schematic illustrates the *MPL* protein, delineating its specific domains: the non-cytoplasmic domain (26–489), the transmembrane (TM) domain (490–513), and the cytoplasmic domain (514–635). It also marks the Erythropoietin receptor (TPO) ligand-binding region (25–128). The diagram features domain representations with symbols indicating gain-of-function mutations (GOFM) with empty circles and loss-of-function mutations (LOFM) with black diamonds; both categories are accentuated in red. Additionally, the 14 identified nsSNPs with blue squares are highlighted; nsSNPs associated with hematological disorders are indicated in green, while others are marked in black.

InterPro, an integrative tool for protein domain and active site recognition, characterizes these domains based on the evaluation of protein families for functional insights. Through this approach, three distinct functional domains of the *MPL* protein were elucidated: the non-cytoplasmic domain spanning amino acids 26 to 489, the transmembrane domain covering amino acids 490 to 513, and, lastly, the cytoplasmic domain ranging from residues 514 to 635. Figure 2 and Figure S1 visually illustrates the *MPL* protein domains highlighted by InterPro and the locations of the 14 identified nsSNPs through various bioinformatic analyses.

### 3.3. Evolutionary Conservation Analysis

Amino acids situated in the conserved domains of proteins play crucial roles in numerous biological processes, underpinning both the structure and function of the protein. Consequently, nsSNPs that introduce point mutations within these conserved areas are more likely to have detrimental effects compared to nsSNPs located in non-conserved regions.

**Figure 3:**
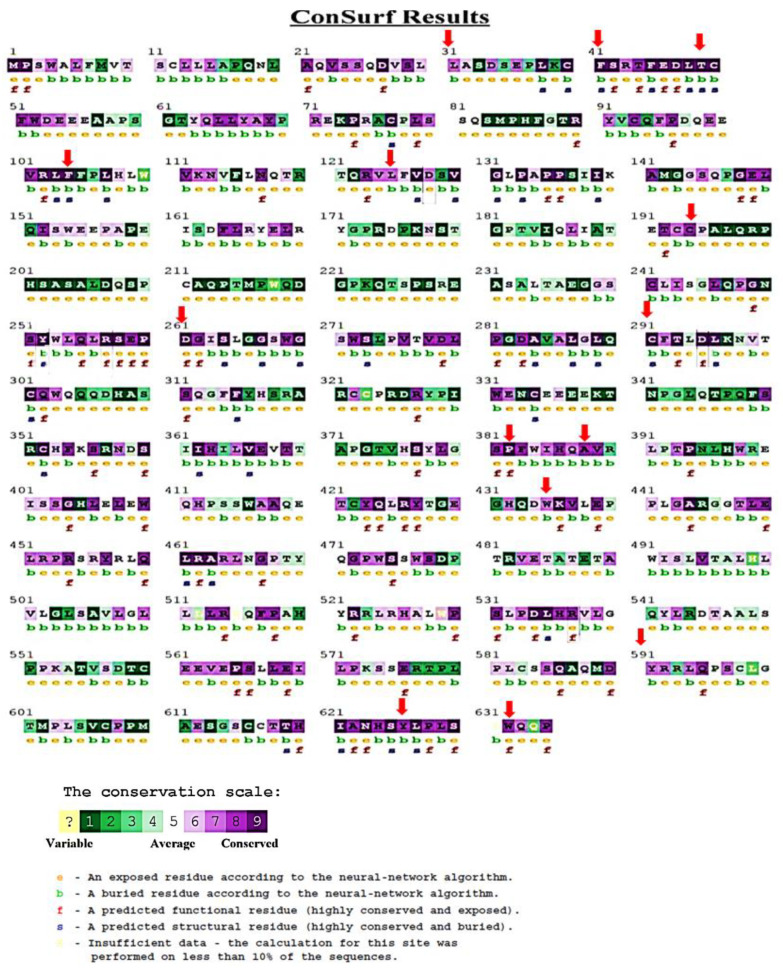
Evolutionary conservation analysis performed for *MPL* protein sequence 1 to 635 aa using ConSurf. Amino acids are shaded according to their conservation ratings and levels. A grade of 1 represents fast-evolving (variable) sites and is marked in turquoise, a grade of 5 denotes sites evolving at a standard rate, shown in white, while a grade of 9 points to (evolutionarily conserved) sites that evolve slowly, represented in maroon. When a particular position covers a range of 4 or fewer color grades, its score is viewed as unreliable. These positions are highlighted in light yellow in the visual display. Question mark means unknown. Red arrows indicate the locations of the identified high-risk nsSNPs.

To determine the evolutionary conservation profiles of the *MPL* protein, we employed the ConSurf web server. This tool specializes in identifying functional and structural amino acids and highlights the evolutionarily conserved residues within proteins. From the 28 previously identified nsSNPs (Table 2) which were deemed likely to be harmful, we analyzed their locations relative to these key conserved domains. Specifically, the nsSNPs causing mutations at the amino acid positions L31P, F41L, T49I, F104S, L125P, C193Y, C291R, A388D, W435C, L510P, Y591D, and Y626S were found within the structural domain. Moreover, mutations at the positions T44I, R257C, D261V, T293N, D295G, P382R, and W632C/R were located within functional domains. All these sites were located in highly conserved regions, receiving scores of 7, 8, or 9 in the ConSurf analysis (as illustrated in Figure 3 and Table 2).

### 3.4. Protein Stability Prediction

Using the I-Mutant 3.0 and MUpro web servers, we evaluated the 21 missense substitutions that had been previously flagged as potentially deleterious. Only those nsSNPs that were unanimously predicted by both tools to negatively impact stability were chosen for subsequent structural assessments. The outcomes of this analysis can be found in Table 3.

### 3.5. Identification of MPL Post-Translational Modification Sites

Utilizing the MusiteDeep server, four residues were identified as potential phosphorylation sites: three were serine-specific, and one was tyrosine-specific. The server further revealed two glycosylation sites, pinpointing locations where carbohydrate molecules could potentially bind as glycosyl donors to hydroxyl groups. These sites were found at tyrosine residues located at positions 180 and 555. In addition, two proline residues were highlighted at positions 2 and 324 as potential hydroxylation sites. The MusiteDeep server also indicated a site at position 516, where Pyrrolidone carboxylic acid might attach to Glutamine residues within MPL. Furthermore, potential methylation could occur on an arginine residue at position 462, while ubiquitination might target a lysine residue at position 339. These post-translational modification (PTM) target sites are detailed in Table 4.

### 3.6. Determination of MPL Protein Interactions

Using the STRING database, a protein association network was constructed for the thrombopoietin receptors. The server employs Gene Ontology to craft a hierarchical structure, providing a comprehensive network analysis that elucidates how the target protein, *MPL*, interfaces with other proteins.

*MPL* is found to associate with four isoforms of the signal transducer and activator of transcription (*STAT*). These include *STAT 1*-alpha/beta and 3 (encoded by *STAT1* and *STAT3* genes) which facilitate cellular reactions to interleukins and various growth factors. *STAT 5A/5B* (encoded by *STAT5A* and *STAT5B* genes) perform dual roles in signal transduction and transcription activation, mediating cellular responses to the cytokine *KITLG/SCF* among others. Furthermore, *MPL* interacts with Tyrosine-protein kinase *JAK2*, a non-receptor tyrosine kinase implicated in diverse cellular activities, from growth and development to differentiation and histone modifications. This kinase plays a pivotal role in both innate and adaptive immune signaling.

Additionally, the thrombopoietin receptors interface with calreticulin, a calcium-binding chaperone encoded by the *CALR* gene, known for promoting protein folding, assembly, and quality assurance within the endoplasmic reticulum via the calreticulin/calnexin cycle.

*MPL* also collaborates with *SHC*-transforming protein 1 (*SHC1*), a signaling adapter linking activated growth factor receptors to specific pathways. The protein forms bonds with Erythropoietin (*EPO*), a hormone pivotal for erythrocyte development, proliferation, and maintenance of circulating erythrocyte mass. Furthermore, *MPL* interacts with Interleukin-3 (*IL3*), a granulocyte/macrophage colony-stimulating factor that plays a role in hematopoiesis by guiding the development and functions of granulocytes and monocytes-macrophages.

**Figure 4:**
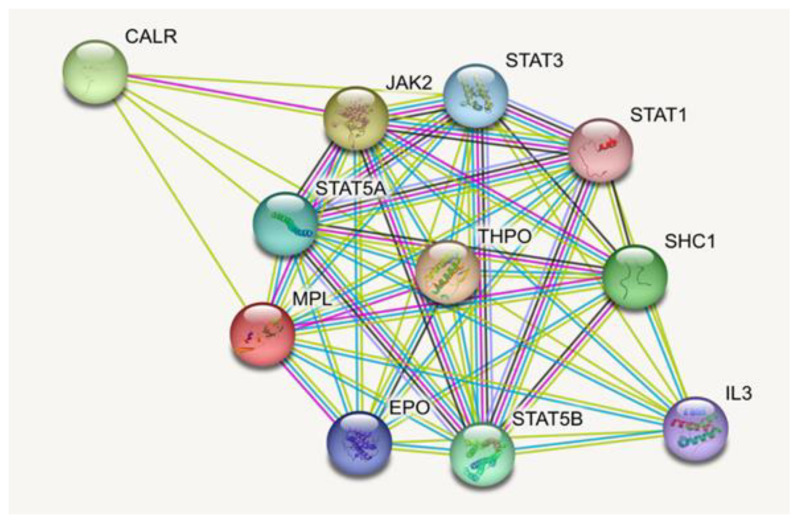
Protein–protein interaction network of *MPL* using a STRING server.

A visual representation of these interactions can be found in Figure 4, while Table S1 offers detailed information on each protein. A Gene Ontology (GO) enrichment analysis of the STRING-derived networks highlights specific roles related to gene mutations, as showcased in Tables S2 and S3.

### 3.7. Evaluation of the Functional Consequences of Non-Coding SNPs

An examination using the Regulome DB database revealed that three SNPs received a score of 2b. Such a score indicates the availability of diverse data types for these nsSNPs, including transcription factor (TF) binding, motif data, DNase Footprint, and DNase peak, specific to their chromosomal positions. Moreover, a score nearing 1 suggests a strong likelihood that these SNPs serve as regulatory variants. The findings from the Regulome DB database analysis are detailed in Table 5.

### 3.8. Project HOPE Analysis

While project HOPE could not produce visual representations due to insufficient structural data, it was noted that the majority of the mutant residues are smaller than the wild-type residues. This size difference is likely to create a void within the protein’s core and result in numerous hydrophobic interactions with molecules present on the protein’s surface. Table 6 shows the project HOPE analysis.

### 3.9. Molecular Mechanism of Pathogenicity Prediction

The 21 identified nsSNPs were assessed using the MutPred2 server to determine their potential impact on protein stability and the molecular consequences of these mutations. Out of these, 13 nsSNPs yielded results that fell within the acceptable threshold range. The mutations’ anticipated functional implications encompassed changes in stability, DNA strand loss, alterations in metal binding, the introduction of disulfide linkages, loop loss, and modifications in transmembrane proteins. A comprehensive breakdown of these results and their corresponding prediction scores can be found in Table 7. It is worth noting that in MutPred2, the score represents an average derived from all neutral networks. A score surpassing a threshold of 0.05 is indicative of potential pathogenicity.

### 3.10. Oncogenicity Determination

To ascertain the oncogenic potential of the identified nsSNPs, we employed CScape and CScape-somatic tools. CScape boasts a 92% accuracy in forecasting the oncogenic attributes of somatic point mutations within the coding regions of the cancer genome. Inputs are structured as per the GRCh38 assembly, encompassing details like chromosome, position, reference, and mutation. Resultant predictions are given in the form of *p*-values spanning 0 to 1. *p*-values above 0.5 are deemed detrimental, while those below 0.5 are considered benign.

CScape-somatic, in contrast, discerns between mutations likely to drive cancer and those merely tagging along, termed passenger mutations. These passenger mutations, often displaying little to no oncogenic properties, accumulate predominantly during the latter stages of tumor development. While the input method for CScape-somatic mirrors that of CScape, it adheres to the GRCh37 assembly. Similar to its counterpart, CScape-somatic offers predictions as *p*-scores ranging between 0 and 1. *p*-scores surpassing 0.5 suggest that the mutation is oncogenic, while those hitting the mark of 0.5 are predicted to be passenger mutations. Detailed findings are cataloged in Table 8.

## 4. Discussion

The *MPL* gene, located on chromosome 1p34.2, is essential for megakaryopoiesis and platelet production through the JAK-STAT signaling pathway, with dysfunctions potentially causing severe thrombocytopenia or thrombocytosis. Furthermore, TPO-MPL signaling can also indirectly promote angiogenesis, primarily through its influence on HSCs and the bone marrow microenvironment [9]. Mutations in the *MPL* gene can lead to hematological disorders, with GOFM associated with malignancies and LOFM causing thrombocytopenia and bone marrow failure [31]. The aim of the current study is to identify and analyze harmful nsSNPs in the *MPL* gene, focusing on their effects on protein structure, function, stability, and hematological disease linkage.

Our study revealed that, of the 635 nsSNPs investigated, 28 were deemed significantly pathogenic by the PredictSNP tool. To understand the locational context of these nsSNPs within *MPL*, we leveraged the InterPro program. This tool demarcated three essential functional domains of *MPL*: the non-cytoplasmic domain, the transmembrane, and the cytoplasmic domain [32]. The significance of pinpointing these domains lies in their fundamental roles: nsSNPs within these domains could potentially alter their structure and subsequent functions.

The identified nsSNPs, Y591D/N, W435C, and F104S are recognized *MPL* mutations linked to various hematological conditions such as Essential Thrombocythemia (ET) and Congenital Amegakaryocytic Thrombocytopenia (CAMT) [31,33]. This association suggests that these particular nsSNPs may serve as significant biomarkers for these diseases. Given the critical roles these mutations could play in disease pathogenesis, further investigation is warranted to explore their potential as diagnostic or prognostic markers. This may also shed light on the molecular mechanisms underlying these hematologic disorders, paving the way for targeted therapeutic strategies.

Additional nsSNPs, specifically L31P, F41L, T44I, and T49A, are located in close proximity to the K39N LOFM, which is linked to thrombocytosis [31]. The spatial closeness of these nsSNPs to the K39N mutation hints at potential combined effects or synergistic interactions that may impact *MPL* signaling pathways. It is crucial to conduct further research to determine if these variants influence the clinical manifestation or outcomes in patients with the K39N mutation.

The nsSNPs identified within the *MPL* gene, specifically in its non-cytoplasmic domain, such as L125P, C193Y, D261V, C291S, P382R, and A388D, are interspersed among established *MPL* hotspot mutations known to be associated with CAMT. This arrangement, as illustrated in Figure 2, raises important considerations regarding their potential functional and clinical implications. The spatial distribution of these nsSNPs in close proximity to known mutation hotspots suggests a potentially significant role in the pathophysiology of the disease.

The nsSNPs identified in our study are interspersed among known *MPL* mutations such as non-cytoplasmic domain LOFM including R102C/P, P106L, T119I, P136H/L, S204F/P, E230G, Y252H, R257C/L, and P275T [31]. For instance, the nsSNP D261V is in close proximity to the R257C/L mutation, known for its LOFM properties. This proximity raises the possibility that D261V could similarly influence *MPL* signaling pathways, potentially altering TPO receptor activity.

The spatial arrangement of these nsSNPs suggests potential cooperative or antagonistic interactions with known *MPL* mutations. For example, the C291S variant, located near the R285E mutation, might alter the receptor’s conformation in a way that exacerbates or mitigates the effects of the R285E mutation. Understanding these interactions could provide insights into the diverse molecular mechanisms underlying *MPL*-related pathologies. Clinically, the distribution of these nsSNPs within *MPL* hotspots may have significant implications for disease phenotype and treatment.

Moreover, our predication analysis revealed that the nsSNPs Y262S and W632C/R, located near the LOFM, P635L [31], which is linked to CAMT, might play a role in inhibiting the JAK-STAT signaling pathway. This proximity raises the possibility that these nsSNPs could have a contributory effect in the dysregulation of this crucial signaling pathway.

Our findings add to the growing body of knowledge about *MPL* mutations. They underscore the need for more comprehensive genetic screening in disorders like ET and CAMT. Future studies should focus on functional assays to elucidate the specific impacts of these nsSNPs on *MPL* signaling and their interactions with known mutations.

The degree to which a protein’s evolutionary conservation influences the impact of mutations is critical in assessing mutation severity. Thus, nsSNPs located in highly conserved regions of a protein are typically more prone to be harmful than those in less conserved, variable regions. The classification of highly conserved residues, depending on whether they are situated on the protein surface or embedded in the core, often delineates their role as either structural or functional [22]. The results from ConSurf were illuminating: out of the 19 nsSNPs investigated, 10 (F41L, T49A, F104S, R257C, D261V, C291R, T293N, D295G, A388D, W435C) exhibited a conservation score at the uppermost end of the scale (score 9). Parsing this further, of these ten nsSNPs, four were inferred to be functionally critical owing to their exposed nature, while six were determined to be pivotal for structural considerations given their buried positioning within the protein. The remaining 9 nsSNPs, though not at the highest conservation score, still exhibited significant conservation, reflected in their scores of 7 and 8. This analysis underscores the potential impact of these nsSNPs, suggesting their potential role in altering protein function or structure.

The stability of a protein is paramount not only for maintaining its structural integrity but also for ensuring its functional efficacy [34]. To pinpoint nsSNPs that could potentially undermine *MPL* protein stability, we leveraged the analytical power of two structural bioinformatics tools: I-Mutant2.0 and MUpro. From our set of 20 nsSNPs, a significant majority, precisely 14 (L31P, F41L, T44I, T49A, F104S, L125P, C193Y, P382R, A388D, W435C, Y591D, Y626S, W632C/R), were identified as detrimental to the stability of the protein. This finding is of particular significance when we consider the intrinsic link between protein stability and its three-dimensional structure, which, in turn, determines the protein’s function. When a protein’s stability is compromised, it can lead to a cascade of negative consequences. There is a risk that the protein might misfold, which can pave the way for its premature degradation. In more severe cases, unstable proteins might conglomerate abnormally, leading to protein aggregates that have been implicated in various pathological conditions [35].

Our analysis using the Musitedeep server revealed a plethora of PTM sites at positions (P2, T180, S228, S232, P324, K339, T393, R462, Q516, T555, S585) within the *MPL* protein. These sites, under normal physiological conditions, could undergo specific modifications, such as phosphorylation, glycosylation, ubiquitination, and others, that can influence the protein’s function. However, an intriguing observation from our study was that none of the most deleterious nsSNPs we identified seemed to be located at or in close proximity to these PTM sites.

An in-depth evaluation by Project HOPE on 13 of these nsSNPs brings forth compelling evidence of their potential to disrupt *MPL*’s structure and function. Notably, some nsSNPs, such as L31P, T49A, and L125P, might create voids within the protein core, potentially rendering it unstable. Others, such as F41L, F104S, Y626S, and W632C/R, may result in the loss of key external molecular contacts, thereby impeding protein interactions. The dynamics of hydrophobic interactions, central to protein stability, are disrupted in C193Y, C291R, P382R, A 388D, and Y591D. This could be due to the potential loss of interactions either within the protein’s core or on its surface. Additionally, nsSNPs like P382R, A388D, Y591D, and W632R/C introduce differential charges compared to the wild-type residues. This introduction, or alteration, of charge can set off electrostatic repulsions with ligands or other similarly charged residues, complicating the protein’s interactions. In essence, the observed differences in size, mass, and charge imparted by these nsSNPs can profoundly influence the dynamics of the *MPL* protein’s interactions, both spatially and temporally. These nsSNPs, given their location and the potential changes they introduce, could very well play a defining role in determining the protein’s biological function and its associated pathogenic outcomes.

The architecture of biological processes is underpinned by intricate protein–protein interactions, which are pivotal in maintaining physiological harmony within the body. STRING, a pivotal database, emerges as an indispensable tool to dissect these interactions by analyzing proteins’ structural, functional, and evolutionary attributes and evaluating functional genomics data [36]. In the realm of hematological processes, the *MPL* protein assumes a cardinal role, orchestrating its functions through interactions with an array of proteins, which, when disrupted, can manifest as a plethora of diseases.

The interaction between *CALR* and *MPL* plays a pivotal role in the regulation of thrombopoietin receptor *MPL* and has been associated with the pathogenesis of myeloproliferative neoplasms (MPNs) [37]. While the binding between wild-type *CALR* and *MPL* primarily occurs in the cytoplasm, the mutated *CALR* proteins found in some MPNs interact with *MPL* on the cell surface, leading to *MPL* activation and downstream signaling [37].

Mutant *CALR* is a central driver of the phenotype associated with MPNs, particularly in cases where *JAK2* or *MPL* mutations are absent. Studies have shown that mutant *CALR* specifically binds to the thrombopoietin receptor *MPL* [38]. Importantly, the positive electrostatic charge on the C terminus of mutant *CALR* has been identified as a crucial requirement for activating *JAK-STAT* signaling via *MPL* [39].

A previous study has shed light on the regions of *MPL* that are essential for supporting the activity of mutant *CALR* (*CALR*^MUT^). This study revealed that *CALR*^MUT^ can interact with the full-length *MPL*, as well as with the extracellular and transmembrane segments of *MPL*. However, it does not interact with the intracellular and transmembrane portions of *MPL* [39].

Some of the identified nsSNPs may potentially disrupt or enhance the interaction between *MPL* and *CALR*^MUT^. For instance, if an nsSNP occurs in a region of *MPL* critical for *CALR*^MUT^ binding, it could modulate the strength or specificity of this interaction, impacting downstream signaling events. Other nsSNPs might influence the interaction between *MPL* and wild-type *CALR*. Changes in *MPL* due to these nsSNPs could potentially affect the normal regulation of *MPL* in the cytoplasm by wild-type *CALR*, potentially leading to altered *MPL* folding and localization.

In intriguing scenarios, specific nsSNPs may exhibit differential effects, enhancing binding to *CALR*^MUT^ while reducing binding to wild-type *CALR*, or vice versa. Such differential effects could result in complex regulatory mechanisms, potentially impacting disease phenotypes in MPNs. Understanding how these nsSNPs may affect the interaction between *CALR* and *MPL* is crucial, as it could shed light on the molecular mechanisms underlying MPNs. Changes in this interaction might influence the activation of *JAK-STAT* signaling, disease progression, and the clinical presentation of MPNs.

Overall, the potential impact of nsSNPs on *CALR-MPL* interactions offers valuable insights into the intricate realm of MPNs. Further research is essential to experimentally validate the effects of these nsSNPs on the binding between *MPL* and both wild-type and mutant *CALR*, providing a deeper understanding of their role in the pathogenesis of MPNs. Such insights could eventually lead to novel therapeutic approaches for these disorders.

As previously discussed, the *TPO-MPL* signaling pathway is known to indirectly facilitate angiogenesis, particularly by influencing HSCs and the bone marrow microenvironment [5]. It is conceivable that the nsSNPs identified in our study could potentially modulate this *TPO-MPL* signaling cascade, subsequently impacting angiogenesis. This speculation is grounded in the understanding that alterations in the *MPL* gene, resulting from these nsSNPs, might affect its interaction with *TPO* and, by extension, the downstream signaling processes. A recent study examining angiogenic factors in ET provides context for this hypothesis. This research focused on subgroups delineated by the presence or absence of *JAK2*V617F, *CALR*, and *MPL*-W515 K/L mutations, revealing no significant variances in the levels of Vascular endothelial growth factor A (VEGF-A), soluble vascular endothelial growth factor receptor-1 (sVEGFR-1), and soluble vascular endothelial growth factor receptor 2 (sVEGFR-2) among these groups [40]. Notably, however, patients harboring *CALR*^MUT^ exhibited a nearly threefold increase in platelet-derived growth factor- two B subunits (PDGF-BB) levels and significantly lower The chemokine stromal cell-derived factor 1α (SDF-1α) levels compared to those with *JAK2*V617F mutations. This finding suggests that specific genetic alterations within the *MPL* signaling pathway components can differentially influence angiogenic factor levels.

Building upon this, it can be hypothesized that the nsSNPs in the *MPL* gene may lead to similar or distinct alterations in the angiogenic profile of ET patients. For instance, if these nsSNPs affect *MPL* functionality or its interaction with *TPO*, they could potentially alter the levels of key angiogenic factors, akin to the effects observed in patients with *CALR* mutations. This might manifest as changes in angiogenesis-related processes within the bone marrow microenvironment, possibly influencing disease progression and symptomatology in ET and other related hematological disorders. Future research should aim to elucidate the specific impact of these nsSNPs on *MPL* signaling and its downstream effects on angiogenesis. Such studies would not only validate this speculative hypothesis but also enhance our understanding of the molecular mechanisms underpinning *MPL*-related hematologic disorders, potentially guiding more targeted therapeutic interventions.

Utilizing RegulomeDB, a tool to pinpoint possible regulatory variants, we were able to assess nsSNPs based on their presence in DNAase hypersensitive sites or transcription factor binding sites. Notably, our analysis revealed that rs186807040, rs554711682, and rs144331455 had significant regulatory potential, as indicated by their ‘2b’ ranking in RegulomeDB. These nsSNPs exhibited predictions of transcription binding sites, matched motifs, and were associated with a DNase footprint within a DNase peak. Such information is particularly enlightening when we consider that the dysregulation of gene expression or aberrant transcriptional activity can be at the heart of hematological disorders. Therefore, these identified nsSNPs, with their strong regulatory potential, may play crucial roles in the onset, progression, or manifestation of certain hematological conditions. Future research should focus on elucidating the precise role of these SNPs in hematopoiesis and related pathological states.

The understanding of nsSNPs and their implications on protein functionality is central to the comprehension of hematological disorders. Utilizing the Mutpred2 tool, which is highly acclaimed for its accuracy in predicting pathogenicity. While MutPred2 is a comprehensive tool, it is important to acknowledge that certain mutations, especially those with subtle or complex effects, may elude its prediction capabilities. We found that of the 21 analyzed nsSNPs, a significant 13 (F41L, T49A, F104S, L125P, C193Y, R257C, D261V, C291S, T293N, D295G, P382R, A388D, and W435C) are considered high-risk pathogenic nsSNPs. These variants possess the potential to destabilize proteins and enact profound molecular consequences. Such alterations can manifest in a variety of ways, from changes in protein stability to the loss of DNA strands. More intricate changes include the alteration in metal binding capabilities, gain of disulfide linkages, and the loss of protein loops. Additionally, changes in transmembrane proteins could influence cell signaling and communication, vital processes in hematopoiesis.

Hematological disorders encompass a broad range of diseases, some of which can be malignant and have oncogenic potential. In this context, understanding the implications of specific nsSNPs, especially with respect to oncogenicity, is crucial. Using the predictive capabilities of CScape and CScape-somatic, we have been able to delineate between nsSNPs that merely affect protein stability and function and those with potential oncogenic tendencies. The identified amino acid alterations C291S, T293N, D295G, and W435C, while influential in terms of the protein’s stability and functionality, are deemed as passengers without oncogenic potential. This means that, while they can modify the protein’s activity or structure, they do not necessarily make the protein a driver for cancer.

## 5. Conclusions

In conclusion, this study extends beyond merely predicting the pathogenicity of nsSNPs within the *MPL* gene; it delves into analyzing the alterations in protein properties and structural changes instigated by these nsSNPs. Through rigorous analysis, we have identified 28 high-risk pathogenic nsSNPs within the *MPL* gene, which are likely to significantly influence its function and contribute to the development of hematological disorders. Notably, among these, 13 nsSNPs stand out due to their exceptionally high predicted pathogenic potential, as determined by our use of advanced bioinformatics tools. Our comprehensive characterization of these nsSNPs illuminates their potential roles in diseases such as Essential Thrombocythemia and Congenital Amegakaryocytic Thrombocytopenia, positing them as valuable biomarkers and shedding light on the mechanisms driving these conditions. These insights pave the way for the development of targeted therapeutic strategies and underscore the critical role of personalized medicine in effectively managing hematological disorders. By enhancing our genetic understanding of these diseases, our study contributes significantly to the field and underscores the need for a tailored approach to treatment, taking into account the unique genetic makeup of each patient.

## Figures and Tables

**Table 1:** High-risk nsSNPs identified through six computational prediction programs.

SNP ID	Missense nsSNPs	PredictSNP	MAPP, PhD-SNP, SIFT, SNAP	PolyPhen1 and 2
rs752112087	W4R	Deleterious	Deleterious	Damaging
rs878854771	L31P	Deleterious	Deleterious	Damaging
rs769423189	F41L	Deleterious	Deleterious	Damaging
rs772445486	T44I	Deleterious	Deleterious	Damaging
rs764333753	T49A	Deleterious	Deleterious	Damaging
rs1361805832	E100V	Deleterious	Deleterious	Damaging
rs1196161699	F104S	Deleterious	Deleterious	Damaging
rs759736065	L125P	Deleterious	Deleterious	Damaging
rs765939441	G172V	Deleterious	Deleterious	Damaging
rs1313333589	C193Y	Deleterious	Deleterious	Damaging
rs1353158125	L234P	Deleterious	Deleterious	Damaging
rs587778519	G245R	Deleterious	Deleterious	Damaging
rs121913611	R257C	Deleterious	Deleterious	Damaging
rs776198457	D261V	Deleterious	Deleterious	Damaging
rs1399857330	C291R	Deleterious	Deleterious	Damaging
rs1171488184	C291S	Deleterious	Deleterious	Damaging
rs919046499	T293N	Deleterious	Deleterious	Damaging
rs113696793	D295G	Deleterious	Deleterious	Damaging
rs1308079365	P382R	Deleterious	Deleterious	Damaging
rs1016116228	A388D	Deleterious	Deleterious	Damaging
rs1006158872	W435C	Deleterious	Deleterious	Damaging
rs121913613	W491C	Deleterious	Deleterious	Damaging
rs775078555	W491R	Deleterious	Deleterious	Damaging
rs1362911656	L510P	Deleterious	Deleterious	Damaging
rs766642690	Y591D	Deleterious	Deleterious	Damaging
rs751884662	Y626S	Deleterious	Deleterious	Damaging
rs753170989	W632C	Deleterious	Deleterious	Damaging
rs536317440	W632R	Deleterious	Deleterious	Damaging

**Table 2:** Shows the Amino Acids Conservation Scores, confidence intervals, and Conservation Colors.

POS	SEQ	Score	Color	b/e	Function
4	W	0.023	5	b	
31	L	−0.621	7	b	
41	F	−0.999	9	b	s
44	T	−0.648	7	e	
49	T	−1.072	9	b	s
100	E	−0.339	6	e	
104	F	−1.131	9	b	s
125	L	−0.639	7	b	
172	G	0.026	5	b	
193	C	−0.604	7	b	
234	L	−0.022	5	b	
245	G	0.131	5	e	
257	R	−1.185	9	e	f
261	D	−1.186	9	e	f
291	C	−1.117	9	b	s
293	T	−1.149	9	e	f
295	D	−1.03	9	e	f
382	P	−0.931	8	e	f
388	A	−0.983	9	b	s
435	W	−1.013	9	b	s
491	W	0.169	4	b	
510	L	−0.718	8	b	
591	Y	−0.937	8	b	
626	Y	−0.937	8	b	
632	W	−0.77	8	e	f

Abbreviations: b, buried; e, exposed; s, structural; f, functional; POS, position; SEQ, sequence

**Table 3:** Prediction of change in protein stability using I-Mutant2.0 and MUpro.

Position	WT	New	SVM2	RI	DDG Kcal/mol	SVM3	RI	MUpro	DDG
31	L	P	Decrease	1	−1.19	Large decrease	4	Decrease	−1.9490598
41	F	L	Decrease	5	−1.20	Large decrease	3	Decrease	−0.77557062
44	T	I	Increase	2	−0.20	Large decrease	2		
49	T	A	Decrease	6	−0.73	Large decrease	5	Decrease	−0.72653759
100	E	V	Increase	1	0.08	Large increase	2		
104	F	S	Decrease	9	−1.85	Large decrease	6	Decrease	−1.6842812
125	L	P	Decrease	5	−1.78	Large decrease	6	Decrease	−2.4825562
193	C	Y	Decrease	1	0.6	Large decrease	3	Decrease	−0.45224808
257	R	C	Decrease	4	−1.03	Neutral	0	Decrease	−0.50317989
261	D	V	Decrease	1	0.00	Large increase	1	Decrease	−0.35365066
291	C	S	Decrease	7	−0.78	Large decrease	3	Decrease	−0.98271664
293	T	N	Decrease	2	−0.64	Neutral	1	Decrease	−0.66131688
295	D	G	Decrease	3	−0.58	Neutral	1	Decrease	−1.4247549
382	P	R	Decrease	7	−1.08	Large decrease	3	Decrease	−1.0745581
388	A	D	Decrease	0	−0.4	Large decrease	3	Decrease	−0.81662298
435	W	C	Decrease	8	−1.48	Large decrease	3	Decrease	−1.0563564
510	L	P	Decrease	2	−1.12	Neutral	0	Decrease	−2.109342
591	Y	D	Decrease	3	−0.82	Large decrease	3	Decrease	−1.1085767
626	Y	S	Decrease	6	−1.28	Large decrease	3	Decrease	−1.2188289
632	W	C	Decrease	8	−1.37	Large decrease	6	Decrease	−0.76506391
632	W	R	Decrease	7	−085	Large decrease	4	Decrease	−1.026455

Abbreviations: SVM 2 and 3, Support Vector Machines; RI, Reality Index; DDG, change in Gibbs free energy.

**Table 4:** Post-translational modification sites in *MPL* protein predicted by Musitedeep server.

Amino Acid	Position	Post-Translation Modification Site	Score
P	2	Hydroxylation	0.501
T	180	Glycosylation	0.523
S	228	Phosphorylation	0.524
S	232	Phosphorylation	0.551
P	324	Hydroxylation	0.536
K	339	Ubiquitination	0.53
T	393	Phosphorylation	0.542
R	462	Methylation	0.55
Q	516	Pyrrolidone carboxylic acid	0.527
T	555	Glycosylation	0.506
S	585	Phosphorylation	0.553

**Table 5:** RegulomeDB predicts non-coding nsSNPs in *MPL* protein.

Chromosome Location	dbSNP IDs	Rank	Score
chr1:43803512..43803513	rs539244587	3a	0.78994
chr1:43818460..43818461	rs569647683	3a	0.8738
chr1:43818473..43818474	rs538671145	3a	0.52742
chr1:43818553..43818554	rs186807040	2b	0.82679
chr1:43818558..43818559	rs554711682	2b	0.8288
chr1:43818713..43818714	rs144331455	2b	0.8153
chr1:43819939..43819940	rs570264040	3a	0.85638
chr1:43820116..43820117	rs536844021	3a	0.73862

**Table 6:** Interpretation of the impact of amino acid change on MPL protein structure and stability.

Residue	AA Changes	Structure	AA Properties
L31P	rs878854771	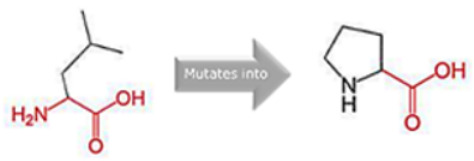	✓ The wild-type and mutant amino acids differ in size. ✓ The mutant residue is smaller than the wild-type residue. ✓ The mutation will cause an empty space in the core of the protein.
F41L	rs769423189	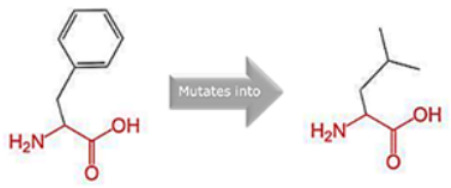	✓ The wild-type and mutant amino acids differ in size. ✓ The mutant residue is smaller than the wild-type residue. ✓ This will cause a possible loss of external interactions.
T49A	rs764333753	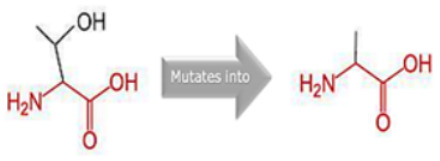	✓ The wild-type and mutant amino acids differ in size. ✓ The mutant residue is smaller than the wild-type residue. ✓ The mutation will cause an empty space in the core of the protein. ✓ The hydrophobicity of the wild-type and mutant residue differs. ✓ The mutation will cause a loss of hydrogen bonds in the core of the protein and, as a result, disturb correct folding.
F104S	rs1196161699	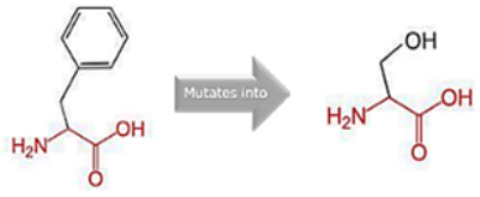	✓ The wild-type and mutant amino acids differ in size. ✓ The mutant residue is smaller than the wild-type residue. ✓ This will cause a possible loss of external interactions. ✓ The hydrophobicity of the wild-type and mutant residue differs. ✓ The mutation might cause a loss of hydrophobic interactions with other molecules on the surface of the protein.
L125P	rs759736065	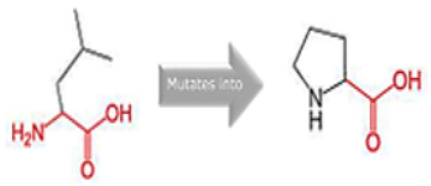	✓ The wild-type and mutant amino acids differ in size. ✓ The mutant residue is smaller than the wild-type residue. ✓ The mutation will cause an empty space in the core of the protein.
C193Y	rs1313333589	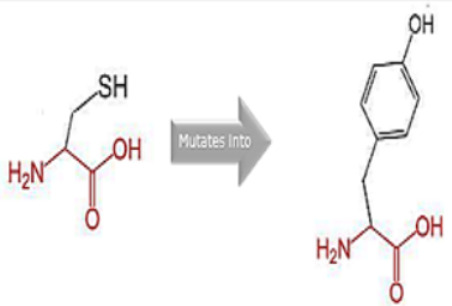	✓ The wild-type and mutant amino acids differ in size. ✓ The mutant residue is bigger; this might lead to bumps. ✓ The hydrophobicity of the wild-type and mutant residue differs. ✓ Hydrophobic interactions, either in the core of the protein or on the surface, will be lost.
C291R	rs1399857330	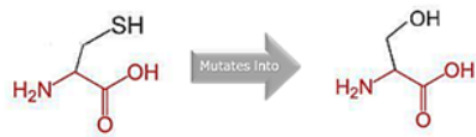	✓ The hydrophobicity of the wild-type and mutant residue differs. ✓ Hydrophobic interactions, either in the core of the protein or on the surface, will be lost.
P382R	rs1308079365	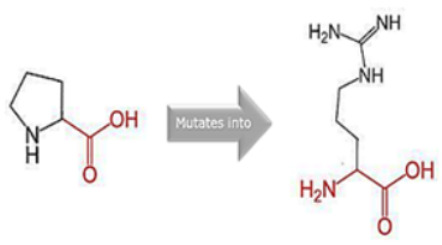	✓ There is a difference in charge between the wild-type and mutant amino acids. ✓ The mutation introduces a charge; this can cause the repulsion of ligands or other residues with the same charge. ✓ The wild-type and mutant amino acids differ in size. ✓ The mutant residue is bigger; this might lead to bumps. ✓ The hydrophobicity of the wild-type and mutant residue differs. ✓ Hydrophobic interactions, either in the core of the protein or on the surface, will be lost. ✓ Prolines are known to have a very rigid structure, sometimes forcing the backbone into a specific conformation. Possibly, your mutation changes a proline with such a function into another residue, thereby disturbing the local structure.
A388D	rs1016116228	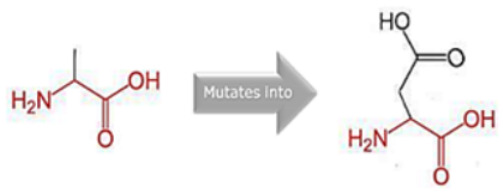	✓ There is a difference in charge between the wild-type and mutant amino acids. ✓ The mutation introduces a charge; this can cause the repulsion of ligands or other residues with the same charge. ✓ The wild-type and mutant amino acids differ in size. ✓ The mutant residue is bigger; this might lead to bumps. ✓ The hydrophobicity of the wild-type and mutant residue differs. ✓ Hydrophobic interactions, either in the core of the protein or on the surface, will be lost.
W435C	rs1006158872	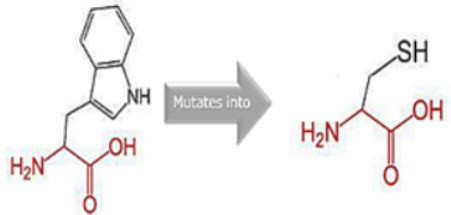	✓ This will cause a possible loss of external interactions. ✓ The wild-type and mutant amino acids differ in size. ✓ The mutant residue is smaller than the wild-type residue.
Y591D	rs766642690	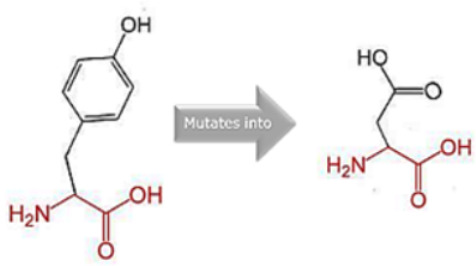	✓ There is a difference in charge between the wild-type and mutant amino acids. ✓ The mutation introduces a charge; this can cause the repulsion of ligands or other residues with the same charge. ✓ The wild-type and mutant amino acids differ in size. ✓ The mutant residue is smaller; this might lead to loss of interactions. ✓ The hydrophobicity of the wild-type and mutant residue differs. ✓ Hydrophobic interactions, either in the core of the protein or on the surface, will be lost.
Y626S	rs751884662	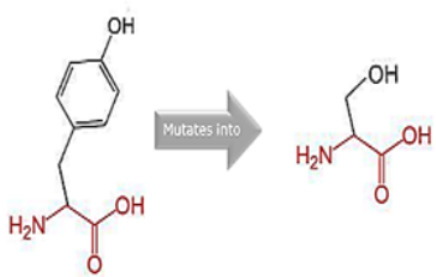	✓ The wild-type and mutant amino acids differ in size. ✓ The mutant residue is smaller, which might lead to loss of interactions.
W632C	rs753170989	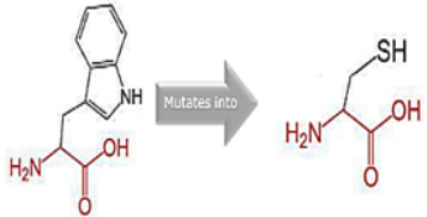	✓ The wild-type and mutant amino acids differ in size. ✓ The mutant residue is smaller; this might lead to loss of interactions.

**Table 7:** The results of the MutPred2 analysis of the 21 nsSNPs, including their MutPred2 score and their impact on different molecular mechanisms.

AA Variation	MutPred2 Score	Molecular Mechanism with *p* Value Less than 0.05
L31P	0.265	-
F41L	0.706	Altered Transmembrane protein Loss of Strand Gain of Disulfide linkage at C40
T44I	0.458	-
T49A	0.752	Altered Metal binding Loss of Strand Altered Transmembrane protein Gain of Disulfide linkage at C50
E100V	0.481	-
F104S	0.922	Altered Ordered interface Altered Metal binding Altered Transmembrane protein Loss of Loop Altered Stability
L125P	0.811	Altered Stability Altered Transmembrane protein Gain of ADP-ribosylation at R120 Loss of Proteolytic cleavage at D128
C193Y	0.562	Loss of Intrinsic disorder Gain of Strand Gain of Loop Altered Transmembrane protein Gain of O-linked glycosylation at T192
R257C	0.750	Altered Transmembrane protein
D261V	0.725	Loss of Loop Altered Transmembrane protein
C291S	0.915	Loss of Disulfide linkage at C291 Altered Transmembrane protein
T293N	0.742	Altered Transmembrane protein Gain of Disulfide linkage at C291 Loss of N-linked glycosylation at N298
D295G	0.808	Altered Transmembrane protein Loss of Disulfide linkage at C291 Loss of N-linked glycosylation at N298
P382R	0.779	Gain of Strand Altered Transmembrane protein
A388D	0.525	Altered Transmembrane protein
W435C	0.790	Altered Metal binding Altered Transmembrane protein Loss of Strand Gain of Disulfide linkage at W435
L510P	0.735	-
Y591D	0.318	-
Y626S	0.324	-
W632C	0.464	-
W632R	0.444	-

**Table 8:** The oncogenic nature of mutations predicted using CScape and CScape-Somatic.

		CScape			CScape-Somatic		
Variant ID	SNP	Input and Assembly GRCh38	Coding Score	Message	Input and Assembly GRCh37	Non-Coding Score	Message
rs769423189	F41L	1,43338140,T,C	0.596320	Oncogenic	1,43338140,T,C	-	-
rs764333753	T49A	1,43338164,A,G	0.626069	Oncogenic	1,43338164,A,G	-	-
rs1196161699	F104S	1,43338640,T,C	0.586258	Oncogenic	1,43338640,T,C	-	-
rs759736065	L125P	1,43338703,T,C	0.872560	Oncogenic	1,43338703,T,C	-	-
rs121913611	R257C	1,43340042,C,T	0.745871	Oncogenic	1,43340042,C,T	-	-
rs776198457	D261V	1,43340055,A,T	0.765347	Oncogenic	1,43340055,A,T	-	-
rs1171488184	C291S	1,43340405,G,C	0.714650	Oncogenic	1,43340405,G,C	0.455926	Passenger
rs919046499	T293N	1,43340411,C,A	0.594031	Oncogenic	1,43340411,C,A	0.223170	Passenger
rs113696793	D295G	1,43340417,A,G	0.869262	Oncogenic	1,43340417,A,G	0.256742	Passenger
rs1308079365	P382R	1,43346609,C,G	0.726814	Oncogenic	1,43346609,C,G	-	-
rs1016116228	A388D	1,43346627,C,A	0.643915	Oncogenic	1,43346627,C,A	-	-
rs1006158872	W435C	1,43346931,G,C	0.853135	Oncogenic	1,43346931,G,C	0.423081	Passenger
rs1362911656	L510P	1,43349323,T,C	0.701219	Oncogenic	1,43349323,T,C	-	-

## Data Availability

Data is contained within the article and Supplementary Material.

## Supplementary Materials

The following supporting information can be downloaded at: https://www.mdpi.com/article/10.3390/cimb45120589/s1, Figure S1. Domain mapping of the MPL protein via InterPro Server. Table S1. Predicted Functional Partners for MPL obtained from the STRING server. Table S2. GO enrichment analysis of the STRING networks created using the core module memberships revealed unique functions in the aging-associated module. Functional enrichments analysis of MPL network. Table S3. RegulomeDB variant classification scheme.
