# Supplementary material for: In Silico Evaluation of Coding and Non-Coding nsSNPs in the Thrombopoietin Receptor (MPL) Proto-Oncogene: Assessing Their Influence on Protein Stability, Structure, and Function
Source: Curr Issues Mol Biol. 2023 Nov 23;45(12):9390–412. doi: 10.3390/cimb45120589 (PMC10742084; doi:10.3390/cimb45120589)
Supplement: Supplementary file 1 [file cimb-45-00589-s001.zip › cimb-2704039-supplementary.pdf]

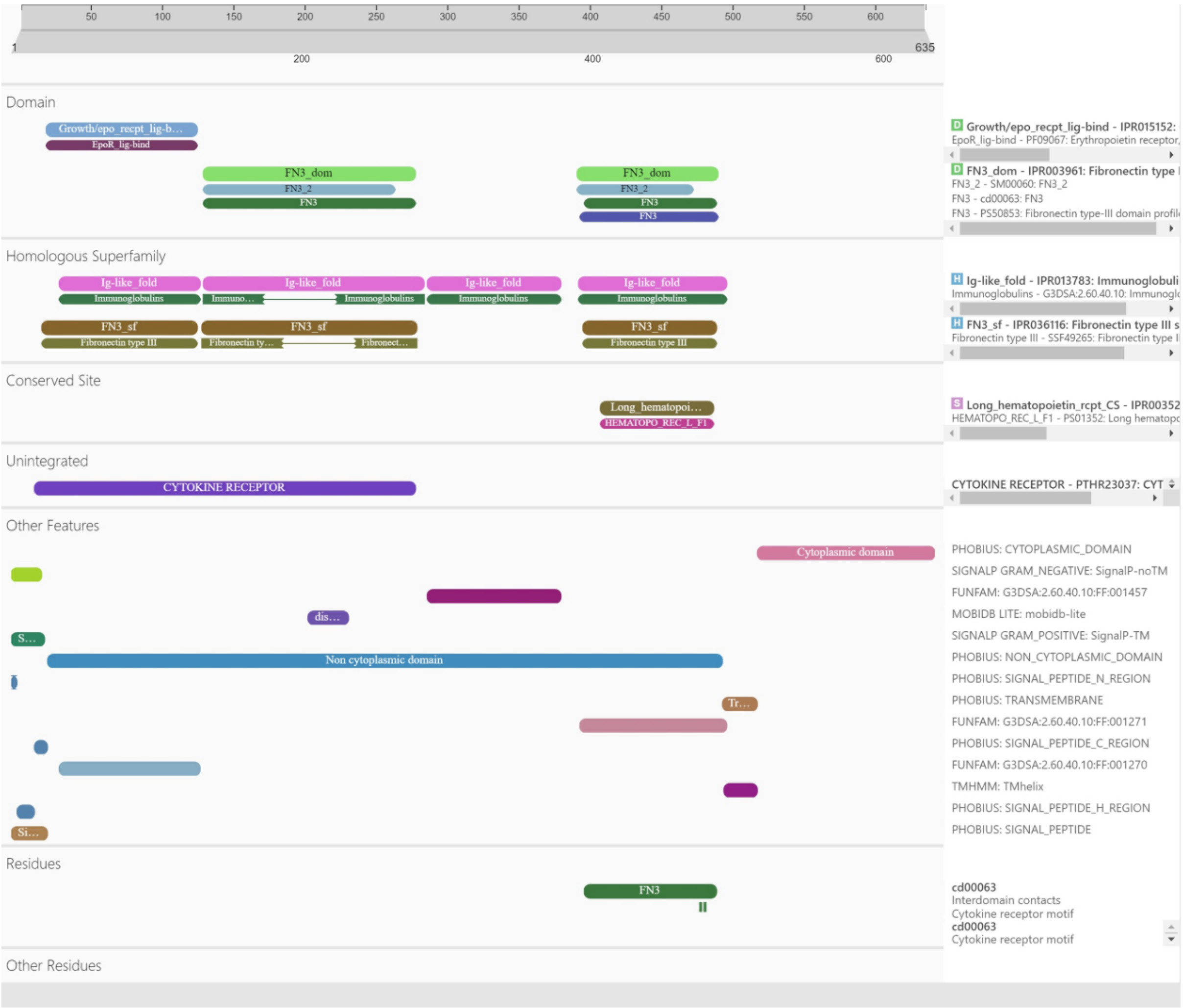

Figure S1. Domain mapping of the *MPL* protein via InterPro Server. This schematic illustrates the *MPL* protein, delineating its specific domains: the non-cytoplasmic domain (26-489), the transmembrane (TM) domain (490-513), and the cytoplasmic domain (514-635).

Table S1. *Predicted Functional Partners for MPL obtained from the STRING server.*

Protein	Information	Score
THPO	P; Lineage-specific cytokine affecting the proliferation and maturation of megakaryocytes from their committed progenitor cells. It acts at a late stage of megakaryocyte development. It may be the major physiological regulator of circulating platelets; Endogenous ligands	0.999
JAK2	Tyrosine-protein kinase JAK2; Non-receptor tyrosine kinase involved in various processes such as cell growth, development, differentiation or histone modifications. Mediates essential signaling events in both innate and adaptive immunity. In the cytoplasm, plays a pivotal role in signal transduction via its association with type I receptors such as growth hormone (GHR), prolactin (PRLR), leptin (LEPR), erythropoietin (EPOR), thrombopoietin (THPO); or type II receptors including IFN-alpha, IFN-beta, IFN-gamma and multiple interleukins. Following ligand-binding to cell surface receptors, [...]	0.998
CALR	Calreticulin; Calcium-binding chaperone that promotes folding, oligomeric assembly and quality control in the endoplasmic reticulum (ER) via the calreticulin/calnexin cycle. This lectin interacts transiently with almost all of the monoglucosylated glycoproteins that are synthesized in the ER. Interacts with the DNA-binding domain of NR3C1 and mediates its nuclear export. Involved in maternal gene expression regulation. May participate in oocyte maturation via the regulation of calcium homeostasis (By similarity)	0.985
SHC1	SHC-transforming protein 1; Signaling adapter that couples activated growth factor receptors to signaling pathways. Participates in a signaling cascade initiated by activated KIT and KITLG/SCF. Isoform p46Shc and isoform p52Shc, once phosphorylated, couple activated receptor tyrosine kinases to Ras via the recruitment of the GRB2/SOS complex and are implicated in the cytoplasmic propagation of mito[illegible]nals. Isoform p46Shc and isoform p52Shc may thus function as initiators of the Ras signaling cascade in various non-neuronal systems. Isoform p66Shc does not mediate Ras activation, [...]	0.983
STAT5B	Signal transducer and activator of transcription 5B; Carries out a dual function: signal transduction and activation of transcription. Mediates cellular responses to the cytokine KITLG/SCF and other growth factors. Binds to the GAS element and activates PRL-induced transcription. Positively regulates hematopoietic/erythroid differentiation	0.967
STAT5A	Signal transducer and activator of transcription 5A; Carries out a dual function: signal transduction and activation of transcription. Mediates cellular responses to the cytokine KITLG/SCF and other growth factors. Mediates cellular responses to ERBB4. May mediate cellular responses to	0.960

	activated FGFR1, FGFR2, FGFR3 and FGFR4. Binds to the GAS element and activates PRL-induced transcription. Regulates the expression of milk proteins during lactation; SH2 domain containing	
STAT3	Signal transducer and activator of transcription 3; Signal transducer and transcription activator that mediates cellular responses to interleukins, KITLG/SCF, LEP and other growth factors. Once activated, recruits coactivators, such as NCOA1 or MED1, to the promoter region of the target gene. May mediate cellular responses to activated FGFR1, FGFR2, FGFR3 and FGFR4. Binds to the interleukin-6 (IL-6)-responsive elements identified in the promoters of various acute-phase protein genes. Activated by IL31 through IL31RA. Acts as a regulator of inflammatory response by regulating differenti [...]	0.954
EPO	Erythropoietin; Hormone involved in the regulation of erythrocyte proliferation and differentiation and the maintenance of a physiological level of circulating erythrocyte mass. Binds to EPOR leading to EPOR dimerization and JAK2 activation thereby activating specific downstream effectors, including STAT1 and STAT3; Endogenous ligands	0.942
IL3	Interleukin-3; Granulocyte/macrophage colony-stimulating factors are cytokines that act in hematopoiesis by controlling the production, differentiation, and function of 2 related white cell populations of the blood, the granulocytes and the monocytes-macrophages; Interleukins	0.934
STAT1	Signal transducer and activator of transcription 1-alpha/beta; Signal transducer and transcription activator that mediates cellular responses to interferons (IFNs), cytokine KITLG/SCF and other cytokines and other growth factors. Following type I IFN (IFN-alpha and IFN-beta) binding to cell surface receptors, signaling via protein kinases leads to activation of Jak kinases (TYK2 and JAK1) and to tyrosine phosphorylation of STAT1 and STAT2. The phosphorylated STATs dimerize and associate with ISGF3G/IRF-9 to form a complex termed ISGF3 transcription factor, that enters the nucleus. ISGF [...]	0.929

Table S2. GO enrichment analysis of the STRING networks created using the core module memberships revealed unique functions in the aging-associated module. Functional enrichments analysis of MPL network

Molecular Function (Gene Ontology)

GO-term	Description	count in network	strength	false discovery rate
GO:0031730	CCR5 chemokine receptor binding	2 out of 7	2.71	0.0067

GO:0035259	Glucocorticoid receptor binding	2 out of 15	2.38	0.0134
GO:0035258	Steroid hormone receptor binding	3 out of 93	1.76	0.0085
GO:0051427	Hormone receptor binding	5 out of 188	1.67	4,20e-05
GO:0016922	Nuclear receptor binding	3 of 114	1.67	0.0134
GO:0035257	Nuclear hormone receptor binding	4 out of 155	1.66	0.0011
GO:0005126	Cytokine receptor binding	6 of 264	1.61	9.19e-06
GO:0070851	Growth factor receptor binding	3 out of 138	1.59	0.0170
GO:0001228	DNA-binding transcription activator activity, RNA polymeras...	4 out of 449	1.2	0.0221
GO:0005102	Signaling receptor binding	9 of 1581	1.01	1.15e-05

Appendix 3 Biology Process (Gene Ontology)

GO-term	Description	count in network	strength	false discovery rate
GO:0008284	Positive regulation of cell population proliferation	11 Out of 919	1.33	3.38e-11
GO:0019221	Cytokine-mediated signaling pathway	10 Out of 678	1.42	1.85e-10

GO:0097696	Receptor signaling pathway via stat	6 Out of 39	2.44	2.05e-10
GO:0007259	Receptor signaling pathway via jak-stat	5 Out of 38	2.37	3.38e-08
GO:0038113	interleukin-9-mediated signaling pathway	4 Out of 9	2.9	6.18e-08
GO:0071310	Cellular response to organic substance	11 Out of 2369	0.92	9.81e-08
GO:0035723	interleukin-15-mediated signaling pathway	4 Out of 13	2.74	1.37e-07
GO:0060397	Growth hormone receptor signaling pathway via jak-stat	4 Out of 14	2.71	1.51e-07
GO:0045639	Positive regulation of myeloid cell differentiation	5 Out of 99	1.95	1.11e-06
GO:0009725	Response to hormone	8 Out of 849	1.22	1.17e-06
GO:0071375	Cellular response to peptide hormone stimulus	6 Out of 269	1.6	1.85e-06
GO:0002682	Regulation of immune system process	9 Out of 1514	1.02	2.72e-06
GO:0032870	Cellular response to hormone stimulus	7 Out of 569	1.34	2.82e-06
GO:0045597	Positive regulation of cell differentiation	8 Out of 993	1.16	3.11e-06
GO:0002376	Immune system process	10 Out of 2481	0.86	4.89e-06

GO:0051240	Positive regulation of multicellular organismal process	9 Out of 1770	0.96	8.25e-06
GO:0050793	Regulation of developmental process	10 Out of 2648	0.83	8.39e-06
GO:0038110	interleukin-2-mediated signaling pathway	3 Out of 11	2.69	1.86e-05
GO:0070106	interleukin-27-mediated signaling pathway	3 Out of 11	2.69	1.86e-05
GO:0070757	interleukin-35-mediated signaling pathway	3 Out of 11	2.69	1.86e-05
GO:0042531	Positive regulation of tyrosine phosphorylation of stat protein	4 Out of 68	2.02	1.89e-05
GO:0048513	Animal organ development	10 Out of 3197	0.75	3.63e-05
GO:0070102	interleukin-6-mediated signaling pathway	3 Out of 16	2.52	3.63e-05
GO:0051239	Regulation of multicellular organismal process	10 Out of 3227	0.74	3.78e-05
GO:0002684	Positive regulation of immune system process	7 Out of 949	1.12	4.62e-05
GO:0050730	Regulation of peptidyl-tyrosine phosphorylation	5 Out of 258	1.54	4.62e-05
GO:0007165	Signal transduction	11 Out of 4876	0.6	5.54e-05

GO:1901700	Response to oxygen-containing compound	8 Out of 1567	0.96	5.54e-05
GO:0030097	Hemopoiesis	6 Out of 570	1.27	6.02e-05
GO:0010604	Positive regulation of macromolecule metabolic process	10 Out of 3600	0.69	9.29e-05
GO:0038111	interleukin-7-mediated signaling pathway	3 Out of 30	2.25	0.00015
GO:0045648	Positive regulation of erythrocyte differentiation	3 Out of 32	2.22	0.00016
GO:0006952	Defense response	7 Out of 1296	0.98	0.00026
GO:1902728	Positive regulation of growth factor dependent skeletal muscle satellite cell proliferation	2 Out of 2	3.25	0.00030
GO:2000026	Regulation of multicellular organismal development	8 Out of 2096	0.83	0.00035
GO:0014070	Response to organic cyclic compound	6 Out of 911	1.07	0.00058
GO:0048584	Positive regulation of response to stimulus	8 Out of 2257	0.8	0.00058
GO:0048872	Homeostasis of number of cells	4 Out o 204	1.54	0.00058
GO:0051173	Positive regulation of nitrogen	9 Out of 3239	0.69	0.00058

	compound metabolic process			
GO:0010628	Positive regulation of gene expression	8 Out o 2337	0.78	0.00071
GO:0007169	Transmembrane receptor protein tyrosine kinase signaling pathway	5 Out of 518	1.23	0.00077
GO:0031325	Positive regulation of cellular metabolic process	9 Out of 3413	0.67	0.00082
GO:0038163	Thrombopoietin-mediated signaling pathway	2 Out of 5	2.85	0.00083
GO:0006950	Response to stress	9 out of 3485	0.66	0.00096
GO:0001934	Positive regulation of protein phosphorylation	6 Out of 1019	1.02	0.0010
GO:0009967	Positive regulation of signal transduction	7 Out of 1654	0.88	0.0010
GO:0031958	Corticosteroid receptor signaling pathway	2 Out of 6	2.77	0.0010
GO:0040014	Regulation of multicellular organism growth	3 Out of 69	1.89	0.0010
GO:0046579	Positive regulation of ras protein signal transduction	3 Out of 69	1.89	0.0010
GO:0010941	Regulation of cell death	7 Out of 1696	0.87	0.0011
GO:0042592	Homeostatic process	7 Out of 1676	0.87	0.0011

GO:0019530	Taurine metabolic process	2 Out of7	2.71	0.0012
GO:0032501	Multicellular organismal process	11 Out of 6933	0.45	0.0013
GO:0038114	interleukin-21-mediated signaling pathway	2 Out o 8	2.65	0.0015
GO:0038155	interleukin-23-mediated signaling pathway	2 Out of 9	2.6	0.0017
GO:0099527	Postsynapse to nucleus signaling pathway	2 Out of 9	2.6	0.0017
GO:0010557	Positive regulation of macromolecule biosynthetic process	7 out of 1906	0.82	0.0021
GO:0051241	Negative regulation of multicellular organismal process	6 Out of 1231	0.94	0.0023
GO:0031328	Positive regulation of cellular biosynthetic process	7 Out of 2005	0.79	0.0028
GO:0002262	Myeloid cell homeostasis	3 Out of 108	1.69	0.0029
GO:0048583	Regulation of response to stimulus	9 Out of 4114	0.59	0.0030
GO:0050867	Positive regulation of cell activation	4 Out of 346	1.31	0.0030
GO:0009966	Regulation of signal transduction	8 Out of 3107	0.66	0.0042

GO:0051770	Positive regulation of nitric-oxide synthase biosynthetic process	2 Out of 16	2.35	0.0042
GO:0030879	Mammary gland development	3 Out of 126	1.63	0.0043
GO:0051171	Regulation of nitrogen compound metabolic process	10 Out of 5836	0.48	0.0043
GO:0001936	Regulation of endothelial cell proliferation	3 Out of 134	1.6	0.0050
GO:0050671	Positive regulation of lymphocyte proliferation	3 Out of 134	1.6	0.0050
GO:0033993	Response to lipid	5 Out of 858	1.02	0.0055
GO:0080090	Regulation of primary metabolic process	10 Out of 6032	0.47	0.0055
GO:0032355	Response to estradiol	3 Out of 142	1.57	0.0056
GO:0045785	Positive regulation of cell adhesion	4 Out of 423	1.23	0.0058
GO:0050729	Positive regulation of inflammatory response	3 Out of 144	1.57	0.0058
GO:0006355	Regulation of transcription, dna-templated	8 Out of 3388	0.62	0.0070
GO:0042981	Regulation of apoptotic process	6 Out of 1550	0.84	0.0070
GO:0031323	Regulation of cellular metabolic process	10 Out of 6239	0.45	0.0073

GO:0001819	Positive regulation of cytokine production	4 Out of 461	1.19	0.0076
GO:0001959	Regulation of cytokine-mediated signaling pathway	3 Out of 163	1.52	0.0077
GO:0042104	Positive regulation of activated t cell proliferation	2 Out of 24	2.17	0.0077
GO:0045893	Positive regulation of transcription, dna-templated	6 Out of 1587	0.83	0.0077
GO:0030522	Intracellular receptor signaling pathway	3 Out of 166	1.51	0.0080
GO:0060334	Regulation of interferon-gamma-mediated signaling pathway	2 Out of 26	2.14	0.0085
GO:0051093	Negative regulation of developmental process	5 Out of 983	0.96	0.0089
GO:0060548	Negative regulation of cell death	5 Out of 999	0.95	0.0094
GO:0065009	Regulation of molecular function	9 Out of 4913	0.51	0.0098
GO:0032101	Regulation of response to external stimulus	5 Out of 1013	0.94	0.0099
GO:0032103	Positive regulation of response to external stimulus	4 Out of 511	1.14	0.0099

GO:0050878	Regulation of body fluid levels	4 Out of 509	1.15	0.0099
GO:2000377	Regulation of reactive oxygen species metabolic process	3 Out of 188	1.45	0.0104
GO:0030335	Positive regulation of cell migration	4 Out of 522	1.13	0.0106
GO:1902533	Positive regulation of intracellular signal transduction	5 Out of 1041	0.93	0.0109
GO:0030154	Cell differentiation	8 Out of 3702	0.58	0.0112
GO:0071407	Cellular response to organic cyclic compound	4 Out of 537	1.12	0.0115
GO:0043410	Positive regulation of mapk cascade	4 Out of 543	1.12	0.0118
GO:0032268	Regulation of cellular protein metabolic process	7 Out of 2693	0.66	0.0128
GO:0022603	Regulation of anatomical structure morphogenesis	5 Out of 1095	0.91	0.0130
GO:0070374	Positive regulation of erk1 and erk2 cascade	3 Out of 209	1.41	0.0130
GO:1902531	Regulation of intracellular signal transduction	6 Out of 1807	0.77	0.0130
GO:1901215	Negative regulation of neuron death	3 Out of 211	1.4	0.0131
GO:0030099	Myeloid cell differentiation	3 Out of 221	1.38	0.0145

GO:0033574	Response to testosterone	2 Out of 39	1.96	0.0147
GO:0006953	Acute-phase response	2 Out of 44	1.91	0.0178
GO:0065008	Regulation of biological quality	8 Out of 404	0.55	0.0181
GO:0007595	Lactation	2 Out of 46	1.89	0.0192
GO:0031347	Regulation of defense response	4 Out of 674	1.02	0.0230
GO:0016310	Phosphorylation	5 Out of 1275	0.84	0.0231
GO:0040008	Regulation of growth	4 Out of 676	1.02	0.0231
GO:0045892	Negative regulation of transcription, dna-templated	5 Out of 1273	0.84	0.0231
GO:0048678	Response to axon injury	2 Out of 51	1.84	0.0231
GO:0007568	Aging	3 Out of 274	1.29	0.0246
GO:0045596	Negative regulation of cell differentiation	4 Out of 728	0.99	0.0291
GO:0006357	Regulation of transcription by rna polymerase ii	6 Out of 2172	0.69	0.0296
GO:0061515	Myeloid cell development	2 Out of 60	1.77	0.0296
GO:0045088	Regulation of innate immune response	3 Out of 301	1.25	0.0308
GO:0045765	Regulation of angiogenesis	3 Out of 303	1.25	0.0312
GO:0080134	Regulation of response to stress	5 Out of 1437	0.79	0.0367
GO:0001776	Leukocyte homeostasis	2 Out of 69	1.71	0.0374
GO:0048545	Response to steroid hormone	3 Out of 328	1.21	0.0381

GO:0060333	Interferon-gamma-mediated signaling pathway	2 Out of 70	1.71	0.0381
GO:0007399	Nervous system development	6 Out of 2371	0.65	0.0439
GO:0048699	Generation of neurons	5 Out of 1551	0.76	0.0488
GO:0030218	Erythrocyte differentiation	2 Out of 82	1.64	0.0490

Table S3. RegulomeDB variant classification scheme

Category scheme

Category	Description
	Likely to affect binding and linked to expression of a gene target
1a	eQTL + TF binding + matched TF motif + matched DNase footprint + DNase peak
1b	eQTL + TF binding + any motif + DNase footprint + DNase peak
1c	eQTL + TF binding + matched TF motif + DNase peak
1d	eQTL + TF binding + any motif + DNase peak
1e	eQTL + TF binding + matched TF motif
1f	eQTL + TF binding/DNase peak
	Likely to affect binding
2a	TF binding + matched TF motif + matched DNase footprint + DNase peak
2b	TF binding + any motif + DNase footprint + DNase peak
2c	TF binding + matched TF motif + DNase peak
	Less likely to affect binding
3a	TF binding + any motif + DNase peak
3b	TF binding + matched TF motif
	Minimal binding evidence
4	TF binding + DNase peak
5	TF binding or DNase peak
6	Motif hit

Lower scores indicate increasing evidence for a variant to be located in a functional region. Category 1 variants have equivalents in other categories with the additional requirement of eQTL information.
